# Mechanisms for Temperature Modulation of Feeding in Goldfish and Implications on Seasonal Changes in Feeding Behavior and Food Intake

**DOI:** 10.3389/fendo.2019.00133

**Published:** 2019-03-07

**Authors:** Ting Chen, Matthew K. H. Wong, Ben C. B. Chan, Anderson O. L. Wong

**Affiliations:** School of Biological Sciences, The University of Hong Kong, Hong Kong, China

**Keywords:** appetite control, feeding behavior, temperature change, leptin and leptin receptor, orexigenic factors, anorexigenic factors, goldfish

## Abstract

In fish models, seasonal change in feeding is under the influence of water temperature. However, the effects of temperature on appetite control can vary among fish species and the mechanisms involved have not been fully characterized. Using goldfish (*Carassius auratus*) as a model, seasonal changes in feeding behavior and food intake were examined in cyprinid species. In our study, foraging activity and food consumption in goldfish were found to be reduced with positive correlation to the gradual drop in water temperature occurring during the transition from summer (28.4 ± 2.2°C) to winter (15.1 ± 2.6°C). In goldfish with a 4-week acclimation at 28°C, their foraging activity and food consumption were notably higher than their counterparts with similar acclimation at 15°C. When compared to the group at 28°C during summer, the attenuation in feeding responses at 15°C during the winter also occurred with parallel rises of leptin I and II mRNA levels in the liver. Meanwhile, a drop in orexin mRNA along with concurrent elevations of CCK, MCH, POMC, CART, and leptin receptor (LepR) transcript expression could be noted in brain areas involved in feeding control. In short-term study, goldfish acclimated at 28°C were exposed to 15°C for 24 h and the treatment was effective in reducing foraging activity and food intake. The opposite was true in reciprocal experiment with a rise in water temperature to 28°C for goldfish acclimated at 15°C. In parallel time-course study with lowering of water temperature from 28 to 15°C, short-term exposure (6–12 h) of goldfish to 15°C could also increase leptin I and II mRNA levels in the liver. Similar to our seasonality study, transcript level of orexin was reduced along with up-regulation of CCK, MCH, POMC, CART, and LepR gene expression in different brain areas. Our results, as a whole, suggest that temperature-driven regulation of leptin output from the liver in conjunction with parallel modulations of orexigenic/anorexigenic signals and leptin responsiveness in the brain may contribute to the seasonal changes of feeding behavior and food intake observed in goldfish.

## Introduction

Temperature change in the environment is a key factor known to affect energy metabolism (1) and body growth in animals (2), and these modulatory effects are partly mediated via regulation of food intake (3). In fish models, circannual rhythm of feeding pattern and food intake has been reported, which is under the influence of environmental cues including seasonal change in water temperature (4). However, the effects of temperature on feeding can be quite variable in different fish species. In general, a rise in water temperature tends to increase food intake, e.g., in salmon (*Salmo salar*) (5), cod (*Gadus morhua*) (6), and flounder *(Pleuronectes americanus*) (7), which can be attributed to the metabolic demand of enhanced body growth caused by activation of the GH/IGF-I axis observed at high temperature (especially during summer) (8–10). Nevertheless, an increase in water temperature can also induce voluntary anorexia in fish species, e.g., in Atlantic salmon (*Salmo salar*), and the phenomenon may be caused by a drop in the peripheral stimulator for feeding, namely ghrelin, in systemic circulation (11). Although central expression of orexigenic/anorexigenic signals modified by temperature change has been documented in fish models, e.g., up-regulation of ghrelin in the brain of Chinese perch (*Siniperca chuatsi*) by temperature rise (12) and elevation of CART expression in the hypothalamus of Atlantic cod (*Gadus morhua*) by low temperature (6), a recent study in Arctic charr (*Salvelinus alpinus*) has revealed that the seasonal changes of NPY, AgRP, POMC, CART, and leptin expressed in brain areas involved in feeding control did not correlate with the annual cycle of feeding reported in the species (13). To date, no consensus has been reached regarding the functional role of orexigenic/anorexigenic signals within the central nervous system (CNS) in the circannual rhythm of feeding observed in fish species.

To unveil the mechanisms underlying temperature modulation of feeding in fish models and their functional implications in seasonal variations in feeding behavior and food intake, goldfish was used as the animal model for our study as (i) it is a representative of cyprinid species, the members of which are commercial fish with high market values in Asian countries, and (ii) the background information for feeding behaviors and appetite control are well-documented in the species (7). In the present study, we sought to address the questions on: (i) Whether the goldfish displays a seasonal change in feeding dependent on water temperature which can be reflected by alterations in feeding behavior and food intake? (ii) Can these feeding responses be induced by short-term and/or long-term manipulation of water temperature? (iii) Can the feeding responses caused by temperature change be explained by parallel modifications of orexigenic/anorexigenic signals expressed in the CNS or in periphery tissues (e.g., in the liver)? Using goldfish adapted to water temperature at different times of the year but maintained under a constant photoperiod, different types of feeding behaviors and food consumption were monitored over an 8-month period covering the transition from summer to winter and correlated to the corresponding change in water temperature. To confirm that the differences in feeding responses observed between the summer and winter months indeed were caused by temperature change, long-term and short-term exposure of goldfish to the “summer temperature” (28°C) and “winter temperature” (15°C) were performed to test if the treatment could mimic the seasonal changes in feeding. To elucidate the mechanisms for feeding control by temperature, parallel measurements of leptin I and II mRNA expression in the liver and transcript levels of NPY, AgRP, orexin, CART, POMC, CCK, MCH, and leptin receptor (LepR) in selected brain areas were also conducted. The results of our study have provided new information on the mechanisms for feeding control by temperature change in the environment which may contribute to the seasonal cycle of food intake observed in goldfish.

## Materials and Methods

### Animal Maintenance and Preparation Prior to Experiments

Goldfish (*Carassius auratus*) with body weight of 28–34 g were acquired from local pet stores and maintained at 20 ± 2°C in well-aerated 700 L tanks (a total of ~300 fish with 50–60 fish/tank in ×5 circular tanks with a diameter of 120 cm and water depth of 60 cm) under a 12-h dark:12-h light photoperiod with regular replacement of water at a rate of ~10% total volume every 48 h using a Gardena® C1060 automatic irrigation system (Gardena, Ulm, Germany). To minimize the influence of reproductive status on feeding, mixed sexes of goldfish during sexual regression were used in our studies. For seasonal change in feeding responses (including food consumption and different types of feeding behaviors), the fish were housed in 200 L tanks (with 20–25 fish/tank in ×4 replica tanks with the dimension of 73 × 60 × 60 cm) in a separate room open to the outside via ventilation vents (to allow for seasonal change in water temperature) but maintained under the same photoperiod setting. For monitoring of feeding responses in our seasonality study and long-term/short-term acclimation experiments to summer (28°C)/winter temperature (15°C), the fish were housed in 20 L “observation tanks” (2 fish/tank with the dimension of 35 × 25 × 20 cm and up to ×8 replica tanks per group for individual experiments) with temperature maintained (± 1°C) by a submerged heater and cooling coil linked with a PolyScience® thermal controller (Preston Industries Inc., Niles, IL). To study the feeding responses after temperature acclimation, goldfish were entrained for 14 days with a “one-meal-per-day” feeding schedule (14) (with food provision of 2% BW/fish at 10:00 a.m. using an automatic feeder) prior to the scoring of feeding behaviors/measurement of food intake. For the studies on target gene expression, goldfish were sacrificed by anesthesia with 50 mg/L MS222 (Sigma-Aldrich, St. Louis, MO) followed by spinosectomy according to the procedures approved by the Committee for Animal Use for Research and Teaching at the University of the Hong Kong.

### Seasonal Change in Feeding and Its Correlation With Water Temperature

Seasonal changes in feeding behavior and food consumption were monitored in goldfish over a period of 8 months (Jul, 2016–Feb, 2017) covering the transition from summer to winter under a 12-h dark:12-h light photoperiod. The period from July to Sept of 2016 covered the summer months with an average water temperature at 28.4 ± 2.2°C, from Sept to Nov of 2016 covered the autumn months with water temperature at 24.0 ± 1.7°C, from Nov to Dec of 2016 covered the early-mid phase of winter with water temperature at 20.4 ± 3.5°C, and Jan–Feb of 2017 covered the peak phase of winter with water temperature at 15.1 ± 2.6°C. After a 14-day entrainment of “one-meal-per-day” feeding schedule, feeding behaviors and food consumption were measured as described previously (14). In this case, different patterns of feeding behaviors were recorded for 2 h using a KPsec VD 714 Surveillance System (Avtech) after introduction of food pellets (TetraMin GmBH, Germany; with ~47% crude protein, ~10% crude oils & fat, ~6% crude fiber and supplements of vitamins and minerals). By the end of the 2-h period, the remains of unconsumed pellets were collected and routinely dried in a 45°C oven for 3–4 days until a constant mass had been acquired. After correction for the loss of soluble components in food pellets (~0.15% by weight), the mass difference of the remains vs. the amount added at the beginning (~2% BW/fish) was used as an index for food intake. Based on the video recorded, the cumulative counts of three types of feeding behaviors observed in goldfish, namely complete feeding/surface foraging, incomplete feeding/food spitting and bottom feeding/bottom foraging, were scored manually in a single-blind manner by the parameters defined by Volkoff and Peter (15). Briefly, complete feeding was defined as the feeding act of engulfing food pellets on water surface in a single foraging movement. Incomplete feeding, in contrast, referred to the “food rejection act” of regurgitation/spitting of food pellet without swallowing. Unlike complete feeding occurred on the water surface, bottom feeding was defined as the feeding act to pick up food pellets/debris sunk to the bottom. In the present study, the data for food intake were also correlated with water temperature in individual experiments conducted at different times of the year using Pearson product-moment correlation analysis.

### Feeding Changes With Long-Term Acclimation to Summer and Winter Temperature

To confirm that seasonal variations in feeding observed were caused by temperature change in the environment, goldfish maintained at 20°C during the autumn period (Sep–Oct, 2017) were divided into two groups and subjected to long-term acclimation for 4 weeks in water tanks maintained at summer temperature (28°C) and winter temperature (15°C), respectively. During the period, the fish were trained with “one-meal-per-day” feeding at 28/15°C and used for the scoring of feeding behaviors and food consumption as described in the preceding section. To examine the mechanisms involved in temperature regulation of feeding behavior and food intake, parallel experiments were also performed to study the effects of a 4-week acclimation at 28°C during the summer (July–Aug, 2016) and 15°C during the winter (Jan–Feb, 2017) on transcript expression of feeding regulators identified in the liver and brain areas involved in feeding control in fish models, including the telencephalon, hypothalamus and optic tectum (7). The long-term acclimation at respective temperatures for the two seasons was conducted to minimize the effect of daily fluctuations of water temperature on target gene expression. After acclimation to the respective temperature, the liver and target brain areas were excised and total RNA and genomic DNA were extracted with Trizol (Invitrogen) according to the instructions of the manufacturer. DNA contents in individual samples were quantified by OD_260/280_ reading and the data obtained were then used for subsequent data normalization for target gene expression. The RNA samples prepared were digested with DNase I, reversely transcribed by Superscript II (Invitrogen), and subjected to real-time PCR for transcript measurement of target regulators for feeding in goldfish using a RotorGene-Q qPCR System (Qiagen) with a Lightcycler® 480 SYBR Green I Master Kit (Roche) (16). PCR reactions were conducted with primers and PCR conditions for different gene targets as shown in Table 1. In our study, parallel measurements of β actin and elongation factor Iα (EF-Iα) gene expression were also conducted to serve as internal controls.

**Table 1:** Primer sequences and PCR conditions for real-time PCR assays.

**Gene target/GenBank accession no**	**PCR condition:**				**Cycle**	***Tm***	**Product size**
**Upstream & downstream primer sequences**	**Denaturing**	**Annealing**	**Extension**	**Detection**			
***β*****-ACTIN/AB039726**							
5′-CTGGTATCGTGATGGACTCT-3′	94°C	56°C	72°C	87°C	×35	91°C	285 bp
5′-AGCTCATAGCTCTTCTCCAG-3′	30 s	30 s	30 s	20 s			
**EF-Iα/AB056104**							
5′-GATTGTTGCTGGTGGTGTTG-3′	94°C	52°C	72°C	87°C	×35	89°C	216 bp
5′-GCAGGGTTGTAGCCGATTT-3′	30 s	30 s	30 s	20 s			
**LEPTIN I/FJ534535**							
5′-TCCAAAGCTCCTCATAGG-3′	94°C	50°C	72°C	86°C	×45	89°C	270 bp
5′-TGGTGGGTGGCGTTTTCC-3′	30 s	30 s	30 s	20 s			
**LEPTIN II/FJ854572**							
5′-TATCGTGGACACCCTAACTAC-3′	94°C	50°C	72°C	85°C	×45	89°C	224 bp
5′-GGTCTAAAGCCAAGAACCCTAA-3′	30 s	30 s	30 s	20 s			
**LEPTIN RECEPTOR/EU911005**							
5′-CTGGCTTGAAGGTGAACGGAC-3′	94°C	65°C	72°C	78°C	×45	87°C	156 bp
5′-TTGGGTGACAGTGCAGTAGTC-3′	30 s	30 s	30 s	20 s			
**CART/AF288810**							
5′-CCAAAGGACCCGAATCTGA-3′	94°C	64°C	72°C	72°C	×35	90°C	171 bp
5′-TTTGCCGATTCTTGACCCT-3′	30 s	30 s	30 s	20 s			
**CCK/CAU70865**							
5′-CCGCAGTCTCAGAAGATGGG-3′	94°C	64°C	72°C	87°C	×35	91°C	197 bp
5′-GGAGGGGCTTCTGCGATA-3′	30 s	30 s	30 s	20 s			
**MCH/AM403730**							
5′-AGGCTTGAGCGAGAACTTGG-3′	94°C	64°C	72°C	86°C	×35	91°C	272 bp
5′-CCCAGAAGACCTACACCTCCC-3′	30 s	30 s	30 s	20 s			
**POMC/AJ431209**							
5′-AAGCGCTCCTACTCCATGGA-3′	94°C	60°C	72°C	83°C	×35	85°C	282 bp
5′-CTCGTCCCAGGACTTCATGAA-3′	30 s	30 s	30 s	20 s			
**NPY/M87297**							
5′-GTAGTGTTGCGGGTAGCGA-3′	94°C	64°C	72°C	88°C	×35	92°C	234 bp
5′-CAGACACCCCGACCCAAG-3′	30 s	30 s	30 s	20 s			
**OREXIN/DQ923590**							
5′-GCAGAGCTGCTCATTGTTGACGTT-3′	94°C	64°C	72°C	84°C	×35	82°C	286 bp
5′-AACCTTGTGATTACCTCAGGAGT-3′	30 s	30 s	30 s	20 s			
**AGRP/AJ555492**							
5′-TGGCATCACATCCAAACCT-3′	94°C	64°C	72°C	82°C	×35	88°C	230 bp
5′-CAGGTGATGACCCAAGCAG-3′	30 s	30 s	30 s	20 s			
**APELIN/FJ755698**							
5′-GAGCATAGCAAAGAGCTGGA-3′	94°C	64°C	72°C	89°C	×35	94°C	340 bp
5′-GCTGAGGATGAGTGGCTTGT-3′	30 s	30 s	30 s	20 s			

### Feeding Responses and Gene Expression Induced by Short-Term Temperature Change

**Figure 1:**
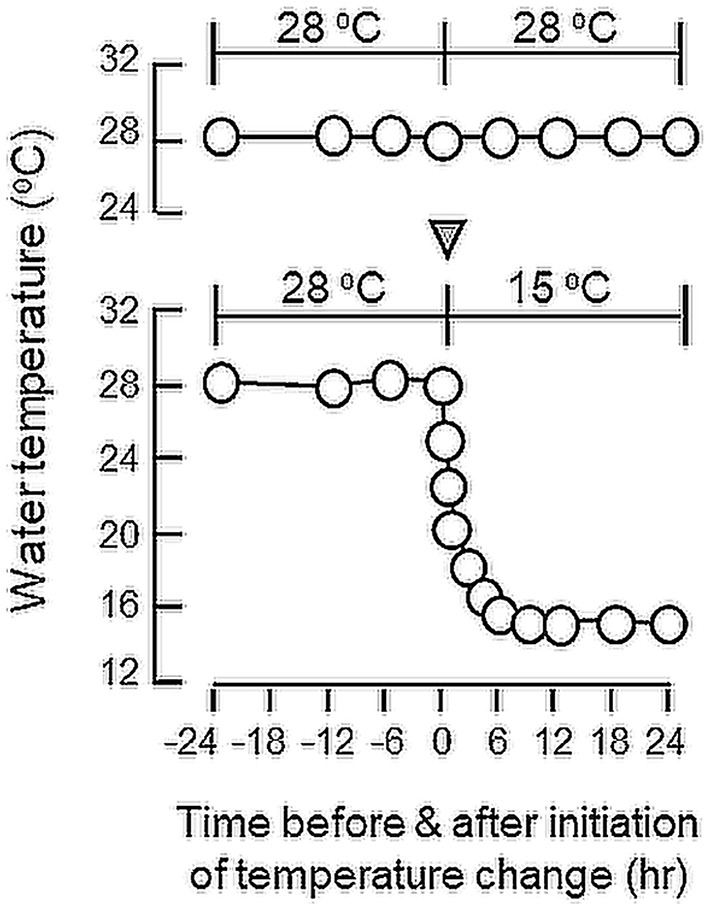
Profile of temperature change during the short-term acclimation of goldfish from summer temperature (28°C) to winter temperature (15°C). Goldfish were maintained in 28°C water for 4 weeks during the summer (Jun–July, 2017) prior to the activation of the cooling system linked to the water tank to gradually reduce the water temperature to 15°C (as indicated by the inverted triangle). The cooling system could allow for a gradual drop in water temperature from 28 to 15°C within 6 h without the need of transferring the fish during the experiment. Without activating the cooling system (as shown in upper panel), water temperature was maintained at 28°C without noticeable change over a 24-h period.

To study the short-term responses induced by temperature change, goldfish trained with “one-meal-per-day” feeding and acclimated at 28°C were transferred to water tanks at 15°C for 24 h. Parallel transfer of goldfish to water tanks at 28°C was used as a control treatment. After 24-h exposure to temperature drop, feeding experiment was initiated (at 28°C for control and 15°C for treatment) to monitor the effects of acute temperature change on feeding behaviors and food consumption as described previously. To test for the reversibility of temperature effect, reciprocal experiment was also performed by transferring goldfish acclimated at 15°C to water tanks at 28°C for 24 h. In this case, parallel transfer to water tanks at 15°C was used as the control group. To establish the time-course of target gene expression for feeding regulators associated with the short-term temperature change, goldfish acclimated at 28°C was housed in water tanks linked with the thermal controlling unit to allow for a gradual drop of water temperature to 15°C in 6 h without disturbing the fish (Figure 1). After that, the fish was maintained at 15°C until the end of the 24-h period. For the control treatment, fish were housed in water tanks at 28°C with no temperature change over the same period. In our study, the liver as well as selected brain areas including the telencephalon, hypothalamus and optic tectum were harvested from individual fish at 24 and 12 h before and at 0, 6, 12, and 24 h after the initiation of temperature drop to 15°C. Total RNA and DNA were extracted from these samples with Trizol and RT samples prepared were then used for real-time PCR measurement of target gene expression as described in the preceding section.

### Data Transformation and Statistical Analysis

For measurement of feeding behaviors, cumulative counts for different types of feeding behaviors were scored every 10 min continuously over a period of 2 h. Food consumption over the 2-h period was normalized as the mass of food pellets taken by the fish over 60 min and used as an index for food intake after thermal acclimation. For real-time PCR of target gene expression, standard curves constructed with serial dilutions of plasmid DNA carrying the ORF/amplicons for target genes with a dynamic range of ≥10^5^, amplification efficiency ≥98% and correlation coefficient ≥0.95 were used for data calibration with RotorGene Q-Rex software (Qiagen). To adjust for variations in the amount of tissues used in RNA extraction, raw data for transcript expression (in femtomole mRNA detected) were expressed as a ratio of genomic DNA (per μg DNA) detected in the same sample. Since the internal controls for β actin and EF-Iα did not show significant difference after long-term/short-term acclimation, the normalized data were presented directly or transformed as a percentage of mean values in the reference control. For the data obtained from seasonality study or experiments with 4-week/24-h acclimation to summer/ winter temperature (with temperature change as the variable), statistical analysis with Student's *t*-test or one-way ANOVA followed by Tukey *post-hoc* test was performed. For the time-course study on gene expression with temperature drop from 28 to 15°C (with time and temperature change as two variables), the data were analyzed by two-way ANOVA prior to Tukey test. In both cases, data presented are expressed as mean ± SEM (*n* = 10–16) and differences between treatment groups were considered as significant at *p* < 0.05.

## Results

### Seasonal Change in Feeding and Its Correlation With Water Temperature

**Figure 2:**
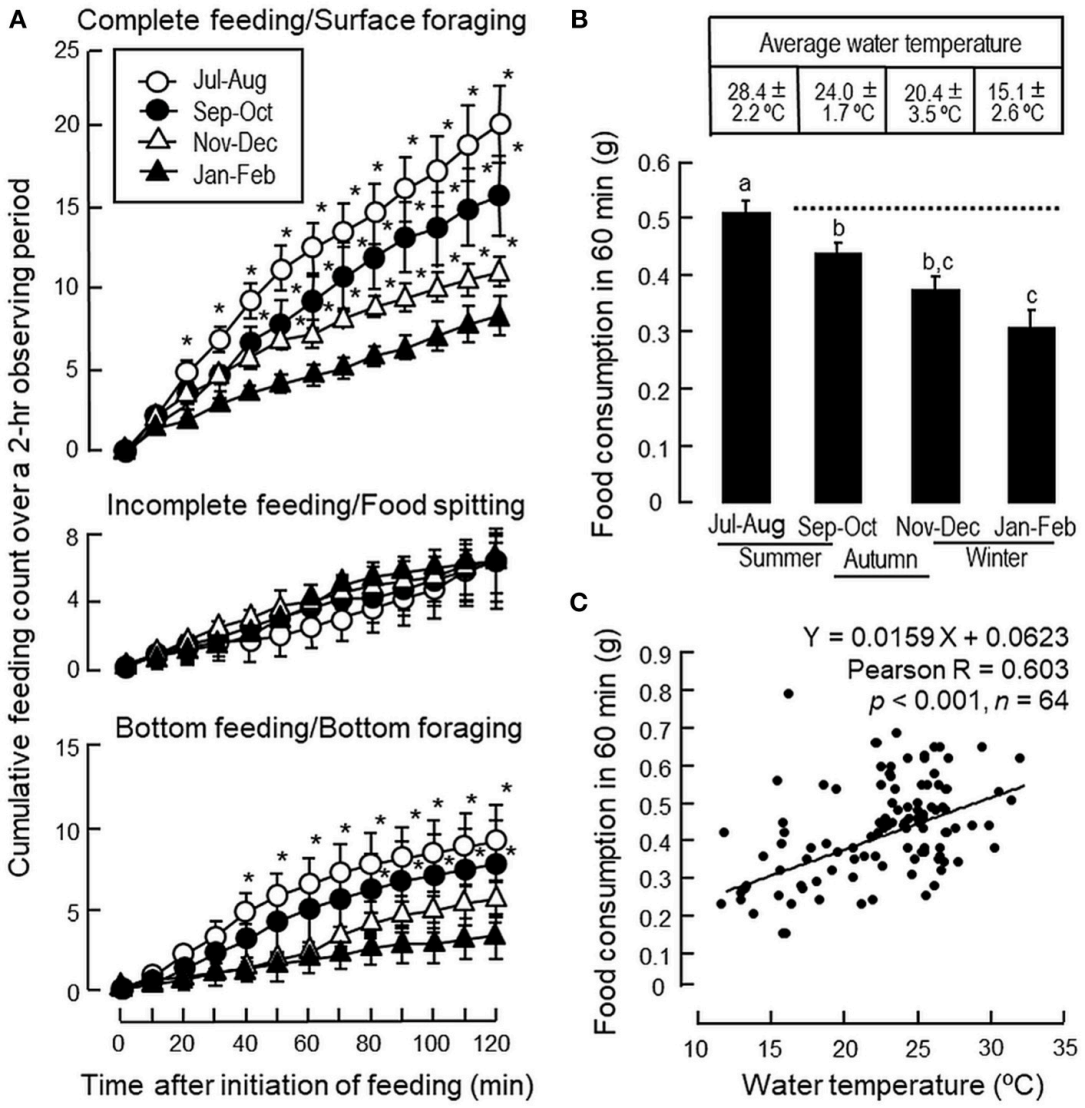
Seasonal changes of feeding behaviors and food intake in goldfish during the transition from summer to winter. **(A)** Seasonal changes of complete feeding/surface foraging, incomplete feeding/food spitting and bottom feeding/bottom foraging during the transition from the summer (Jul–Aug, 2016), autumn (Sep–Oct, 2016), early-mid phase of the winter (Nov–Dec, 2016) to the peak phase of the winter (Jan–Feb, 2017). **(B)** Seasonal change of food consumption related to the temperature drop in the environment during the same period. **(C)** Positive correlation of the gradual decline in food intake observed during the transition from summer to winter months as shown in **(B)** with the parallel drop in water temperature as revealed by Pearson product-moment regression analysis. Data presented, including feeding behaviors, food consumption and water temperature are expressed as mean ± SEM (*n* = 14–16). Feeding behaviors were scored over a period of 2 h and the data of feeding counts obtained during the summer, autumn and early-mid phase of the winter were compared with the corresponding data of the same time point from the group scored during the peak phase of the winter using Student's *t*-test. For food intake occurred during the same period, the data for food consumption from different groups were analyzed by one-way ANOVA followed by Tukey *post-hoc* test. Differences between treatment groups were considered as significant at *p* < 0.05.

In goldfish subjected to seasonal change in temperature during the transition from summer to winter, except for a lack in response for incomplete feeding/food spitting activity, the cumulative counts for feeding behaviors, including complete feeding/surface foraging and bottom feeding/ bottom foraging, were found to be reduced gradually from the summer (Jul–Aug, 2016), autumn (Sept–Oct, 2016), early-mid phase of the winter (Nov–Dec, 2016) to the peak phase of winter (Jan–Feb, 2017) (Figure 2A). During the same period, water temperature was reduced from 28.4 ± 2.2°C in summer to 15.1 ± 2.6°C during the peak phase of winter with a gradual drop in food consumption (Figure 2B). In the same study, Pearson's analysis also revealed a positive correlation between the drop in water temperature and the gradual decline in food consumption during the progression from summer to winter period (Figure 2C).

### Long-Term Thermal Acclimation on Feeding and Gene Expression of Feeding Regulators

**Figure 3:**
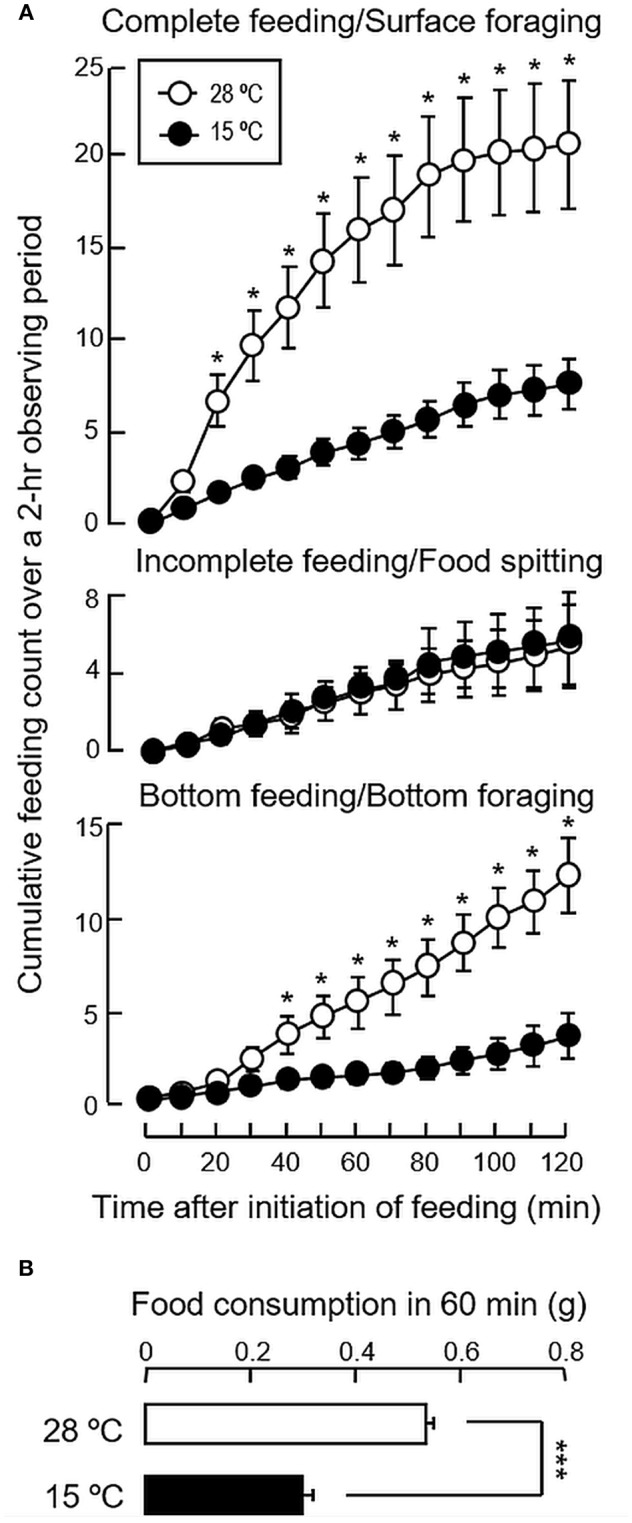
Long-term acclimation to the summer temperature (28°C) and winter temperature (15°C) on feeding behaviors and food consumption in goldfish. Goldfish acclimated to 20°C during the autumn months (Sep–Oct, 2017) were maintained for 4 weeks in 28 and 15°C water tanks respectively prior to the measurement of **(A)** feeding behaviors and **(B)** food consumption. In this experiment, the feeding counts for the three types of feeding behaviors, namely complete feeding, incomplete feeding and bottom feeding, as well as the food intake occurred during the same period were compared between the two groups using Student's *t*-test. Data presented are expressed as mean ± SEM (*n* = 12) and the difference between the two groups was considered as significant at *p* < 0.05 (^*^p < 0.05 and ^***^p < 0.001).

To test if temperature change can serve as the cause for seasonal variations in feeding, long-term acclimation of goldfish for 4 weeks to either summer (28°C) or winter temperature (15°C) were performed. In this case, the cumulative counts for complete feeding/surface foraging and bottom feeding/bottom foraging in the group acclimated at 28°C were found to be notably higher than the group maintained at 15°C (Figure 3A). Similar to the results of seasonal change in feeding behaviors, the counts for incomplete feeding/food spitting were not affected by variation in water temperature. When compared to the group at 28°C, a parallel drop in food consumption was also noted with thermal acclimation to 15°C (Figure 3B), which was in agreement with the decline in foraging activity occurring both at the surface and bottom levels.

**Figure 4:**
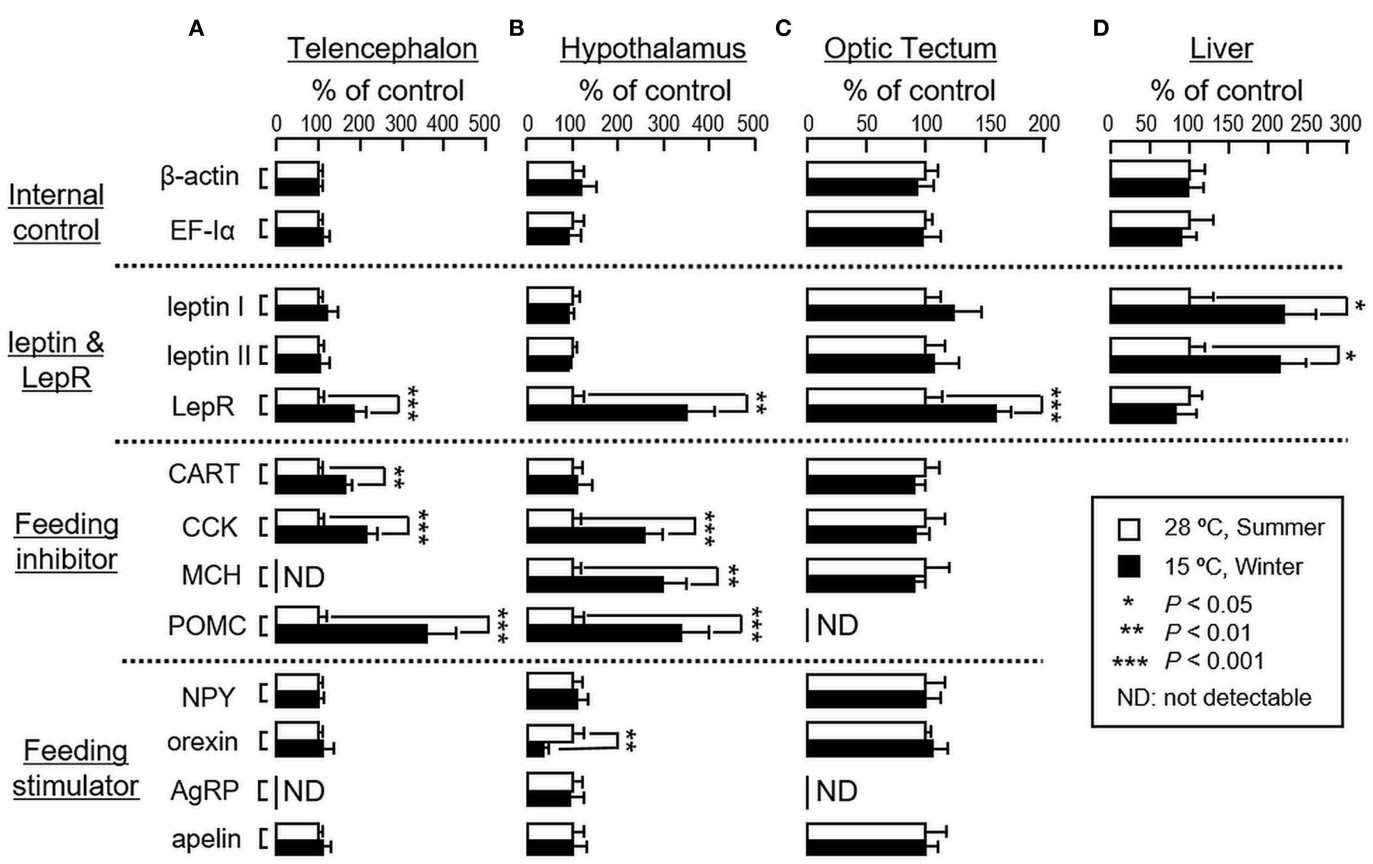
Transcript expression of orexigenic and anorexigenic factors in the liver and brain areas involved in feeding control in goldfish during the summer and winter months. To avoid the variability of daily fluctuation in water temperature, goldfish were maintained for 4 weeks at 28°C during the summer (July–Aug, 2016) and at 15°C during the winter (Jan–Feb, 2017). After that, the liver and brain areas, including the telencephalon, hypothalamus and optic tectum, were harvested and used for RNA isolation. RT samples were then prepared and used for real-time PCR for the respective gene targets. In this experiment, parallel measurement of β actin and EF-Iα mRNA expression were also conducted to serve as the internal control. Data presented (mean ± SEM, *n* = 12) were compared with Student's *t*-test and the difference between the two groups was considered as significant at *p* < 0.05 (^*^p < 0.05, ^**^p < 0.01 and ^***^p < 0.001).

In parallel study using goldfish acclimated at 28°C during the summer as a reference control, acclimation of the fish to 15°C during the winter did not alter transcript expression of β actin and EF-Iα in the liver as well as in brain areas including the telencephalon, hypothalamus and optic tectum (Figure 4). In the telencephalon, however, parallel rises in LepR, CART, CCK and POMC mRNA levels were noted with no significant changes in transcript expression for leptin I, leptin II, NPY, orexin and apelin (Figure 4A). A similar pattern of transcript expression was also observed in the hypothalamus except that 15°C acclimation during winter did not alter CART expression but induced an elevation in MCH with a concurrent drop in orexin mRNA level (Figure 4B). In the optic tectum, unlike the responses in telencephalon/hypothalamus, except for the rise in LepR mRNA, significant changes in transcript expression for the other target genes examined were not apparent (Figure 4C). In the same study, interestingly, acclimation at 15°C during the winter was effective in increasing leptin I and II mRNA levels in the liver but with no concurrent change in LepR gene expression at the hepatic level (Figure 4D).

### Short-Term Thermal Acclimation on Feeding and Gene Expression of Feeding Regulators

**Figure 5:**
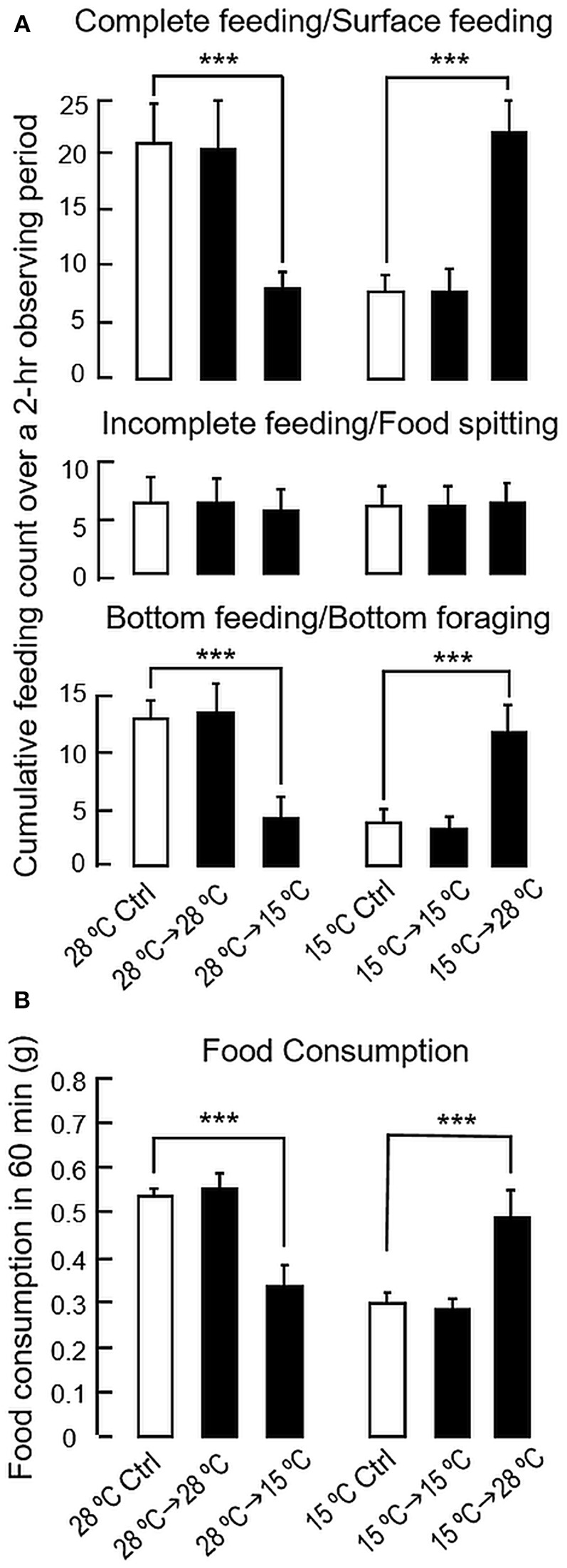
Short-term acclimation to the summer temperature (28°C) and winter temperature (15°C) on feeding behaviors and food consumption in goldfish. Goldfish acclimated to 20°C during the autumn months (Sep–Oct, 2017) were maintained for 4 weeks in 28 and 15°C water tanks, respectively. After that, the fish acclimated to 28°C were transferred to water tanks at 15°C for 24 h. In reciprocal experiment, the fish acclimated to 15°C were transferred to water tanks at 28°C during the same period. As control treatment, parallel experiments without transferring the fish or with parallel transfer into water tanks with the same acclimation temperature (i.e., from 28 to 28°C/from 15 to 15°C) were also conducted. Following the short-term exposure to temperature change, measurement of different types of feeding behaviors **(A)** and food intake **(B)** were performed according to the standard protocols. The data obtained (mean ± SEM, *n* = 10–12) were analyzed with one-way ANOVA followed by Tukey *post-hoc* test. Difference between groups was considered as significant at *p* < 0.05 (^***^p < 0.001).

As shown in Figure 5A, a notable reduction in the counts for complete feeding/surface foraging and bottom feeding/bottom foraging was observed following a 24-h exposure to 15°C water in goldfish previously acclimated at 28°C, while the opposite was true with parallel transfer of goldfish acclimated at 15°C to 28°C water for 24 h in the reciprocal experiment. Consistent with the results for long-term acclimation, short-term changes in water temperature (from 28 to 15°C/from 15 to 28°C for 24 h) were not effective in altering incomplete feeding/food spitting activity. Of note, modifications in foraging activity were also reflected by corresponding changes in food intake. In this case, food consumption was reduced in 28°C fish after transfer to 15°C water but increased in 15°C fish after transfer to 28°C water (Figure 5B). In contrast, parallel transfer of goldfish to water tanks with “acclimated temperature” (i.e., 28°C to 28°C and 15°C to 15°C) did not trigger any noticeable changes in feeding behaviors/food intake, indicating that the feeding responses observed were not caused by handling stress during the experiment.

**Figure 6:**
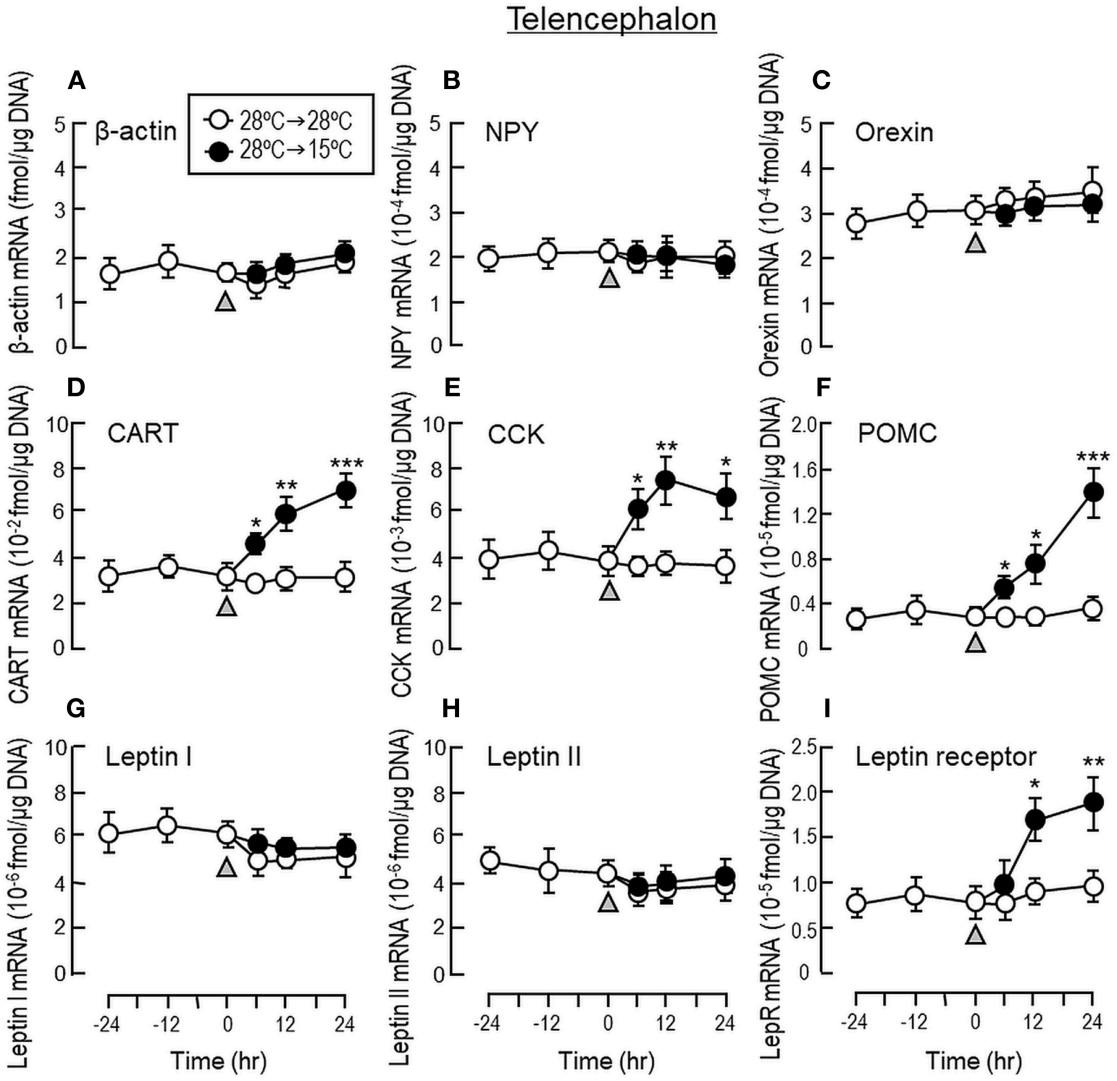
Transcript expression of orexigenic and anorexigenic factors within the telencephalon of goldfish with short-term exposure to winter temperature (15°C). Water temperature for goldfish acclimated at 28°C was gradually reduced to 15°C over a 24-h period using a cooling system linked with the water tank. The telencephalon was harvested from individual fish at different time points before and after the activation of the cooling system (as indicated by gray triangle). Total RNA was isolated, reversely transcribed and used for real-time PCR for respective gene targets, including **(A)** β actin, **(B)** NPY, **(C)** Orexin, **(D)** CART, **(E)** CCK, **(F)** POMC, **(G)** leptin I, and **(H)** leptin II and **(I)** leptin receptor. Parallel experiment with goldfish maintained at 28°C water without activation of the cooling system was used as the control treatment. Similar to the previous study on seasonality of orexigenic/anorexigenic signals, transcript expression of β actin was used as the internal control. For our time course study, the data obtained (mean ± SEM, *n* = 12) were analyzed using two-way ANOVA followed by Tukey test. Difference between groups was considered as significant at *p* < 0.05 (^*^p < 0.05, ^**^p < 0.01, and ^***^p < 0.001).

**Figure 7:**
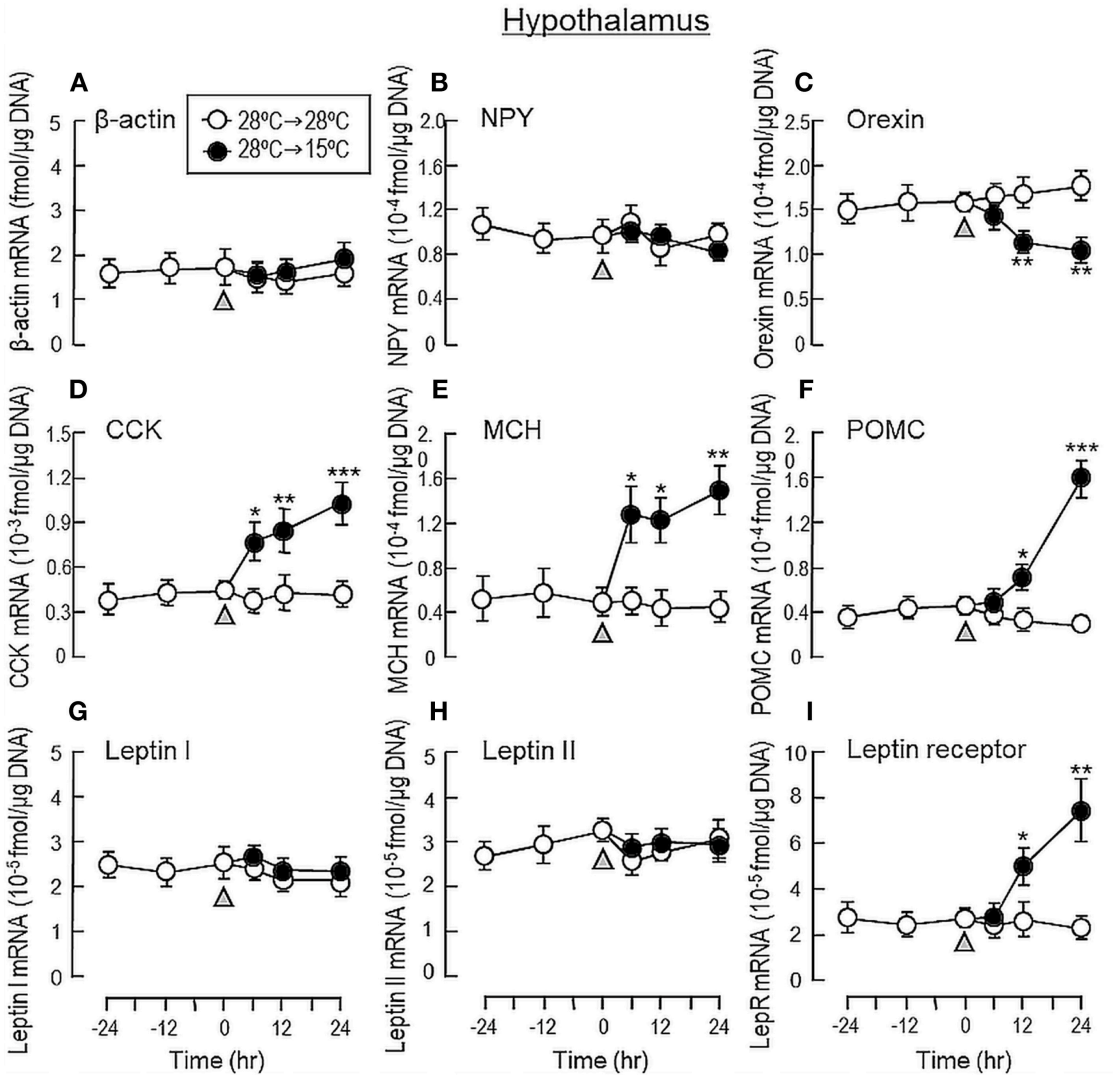
Transcript expression of orexigenic and anorexigenic factors within the hypothalamus of goldfish with short-term exposure to winter temperature (15°C). Water temperature for goldfish acclimated at 28°C was gradually reduced to 15°C over a 24-h period using a cooling system linked with the water tank. The hypothalamus was harvested from individual fish at different time points before and after the activation of the cooling system (as indicated by gray triangle). Total RNA was isolated, reversely transcribed and used for real-time PCR for respective gene targets, including **(A)** β actin, **(B)** NPY, **(C)** Orexin, **(D)** CCK, **(E)** MCH, **(F)** POMC, **(G)** leptin I, and **(H)** leptin II and **(I)** leptin receptor. Parallel experiment with fish maintained at 28°C water without activation of the cooling system was used as the control treatment. For our time course study, the data obtained (mean ± SEM, *n* = 12) were analyzed with two-way ANOVA followed by Tukey test. Difference between groups was considered as significant at *p* < 0.05 (^*^p < 0.05, ^**^p < 0.01, and ^***^p < 0.001).

**Figure 8:**
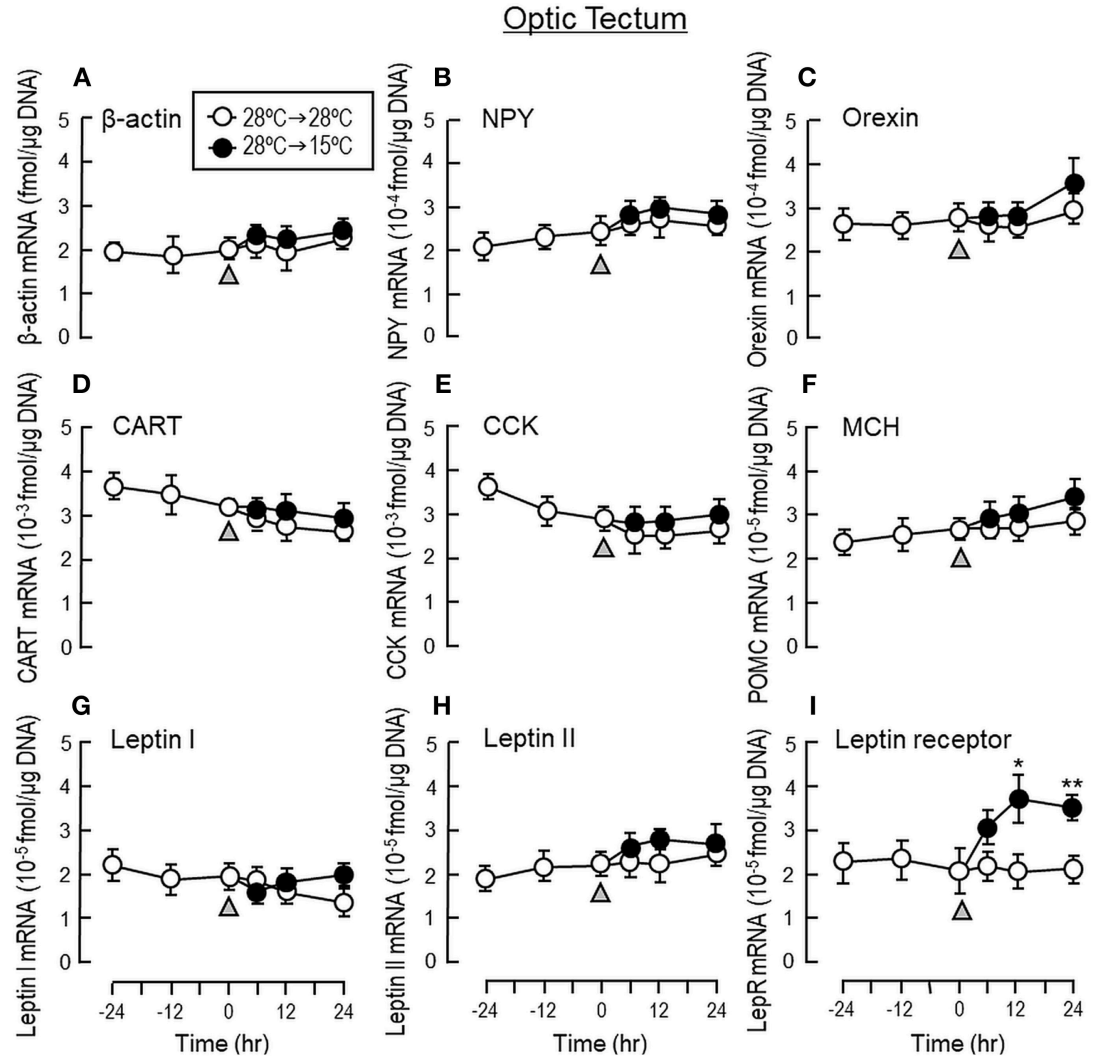
Transcript expression of orexigenic and anorexigenic factors within the optic tectum of goldfish with short-term exposure to winter temperature (15°C). Water temperature for goldfish acclimated at 28°C was gradually reduced to 15°C over a 24-h period using a cooling system linked with the water tank. The optic tectum was harvested from individual fish at different time points before and after the activation of the cooling system (as indicated by gray triangle). Total RNA was isolated, reversely transcribed and used for real-time PCR for respective gene targets, including **(A)** β actin, **(B)** NPY, **(C)** Orexin, **(D)** CART, **(E)** CCK, **(F)** MCH, **(G)** leptin I, and **(H)** leptin II and **(I)** leptin receptor. Parallel experiment with goldfish maintained at 28°C water without activation of the cooling system was used as the control treatment. For our time course study, the data obtained (mean ± SEM, *n* = 12) were analyzed with two-way ANOVA followed by Tukey test. Difference between groups was considered as significant at *p* < 0.05 (^*^p < 0.05, ^**^p < 0.01, and ^***^p < 0.001).

**Figure 9:**
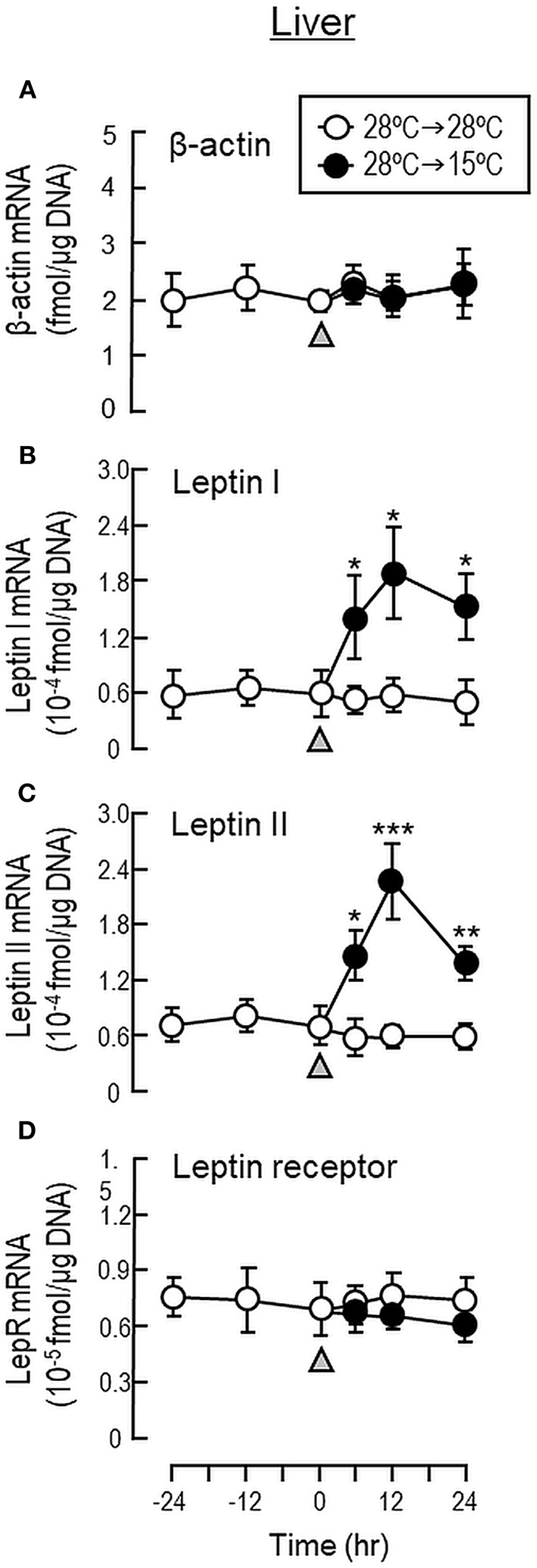
Transcript expression of leptin and leptin receptor in the liver of goldfish with short-term exposure to winter temperature (15°C). Water temperature for goldfish acclimated at 28°C was reduced to 15°C over a 24-h period using a cooling system linked with the water tank. The liver was harvested from individual fish at different time points before and after the activation of the cooling system (as indicated by gray triangle). Total RNA was isolated, reversely transcribed and used for real-time PCR for respective gene targets, including **(A)** β actin, **(B)** leptin I, **(C)** leptin II and **(D)** leptin receptor. Parallel experiment with goldfish maintained at 28°C water without activation of the cooling system was used as the control treatment. For our time course study, the data obtained (mean ± SEM, *n* = 12) were analyzed with two-way ANOVA followed by Tukey test. Difference between groups was considered as significant at *p* < 0.05 (^*^p < 0.05, ^**^p < 0.01, and ^***^p < 0.001).

To shed light on the mechanisms for feeding control by short-term temperature change, a time-course experiment was conducted in goldfish acclimated at 28°C with a gradual drop of water temperature from 28°C to 15°C. In our system, water temperature could be reduced to 15°C within the first 6 h after initiation of temperature change (Figure 1). Similar to our seasonality study, short-term exposure to 15°C could trigger differential changes in transcript expression of feeding regulators in the liver as well as in different brain areas. In the telencephalon, CART, CCK, POMC and LepR mRNA levels were found to be elevated in a time-dependent manner with no significant changes in β actin, NPY, orexin, leptin I and leptin II gene expression (Figure 6). The pattern of transcript expression in the hypothalamus, including the rises in CCK, POMC, and LepR gene expression, was comparable with that of the telencephalon. Interestingly, a drop in orexin mRNA with a parallel rise in MCH transcript level were also noted, which were absent in the telencephalon (Figure 7). In the optic tectum, except for the rise in LepR mRNA, no significant changes were observed regarding the gene expression for β actin, NPY, orexin, CART, CCK, MCH, leptin I, leptin II, and LepR (Figure 8). In the same study, however, leptin I and II mRNA levels were found to be elevated in the liver but without parallel change in β actin and LepR gene expression (Figure 9).

## Discussion

In poikilotherms, especially in fish species, body functions including somatic growth (8, 9, 17), reproduction (18, 19), metabolism (20), locomotor activity (21), stress responses (22), embryonic development (23), and immune functions (24) are known to be sensitive to temperature change. In fish models, circannual cycle in feeding pattern/food intake has been reported and can be associated with seasonal changes in water temperature and photoperiod (4). In general, elevation in feeding can be noted in fish species during the spring/summer months with higher temperature (25). This is at variance with the case in non-hibernating homeotherms, e.g., domesticated cats, with increased feeding in the late autumn/winter (26), which may be related to the elevated metabolic demand for thermogenesis at low temperature. The seasonal change in feeding observed in fish species is also in agreement with the results of previous studies showing that food intake can be reduced by low temperature, e.g., in catfish (*Ictalurus punctatus*) (27), halibut (*Hippoglossus hippoglossus*) (28), sickleback (*Gasterosteus aculeatus*) (29), turbot (*Scophthalmus maximus*) (30), and tench (*Tinca tinca*) (31). However, species-specific variations in feeding responses do exist in fish models. For examples, high temperature is known to induce voluntary anorexia in Atlantic salmon (*Salmo salar*) (11) and summer fasting can also be observed in some cold water fish, e.g., in cunner (*Tautogolabrus adspersus*) (32), suggesting that the “temperature effect” on feeding can be quite different between warm water and cold water species.

To confirm that seasonal change in feeding do exist in goldfish, a cyprinid species known to be well-adapted to a wide range of water temperature, its feeding behavior and food consumption were monitored over a period of 8 months covering the transition from summer to winter. In our study, a gradual decline in foraging behavior (both surface and bottom foraging) was noted during the progression from summer to winter with a parallel drop in water temperature. The decline in foraging activity also occurred with parallel reduction in food intake, which was found to have a positive correlation with the attenuation in water temperature during the same period, suggesting that the seasonal change in environmental temperature may contribute to the observed differences in feeding responses between the summer and winter months. In goldfish, regulation of food consumption can be achieved by alteration of foraging activity in water surface/at bottom level with concurrent modification in food spitting activity, e.g., after treatment with NPY (33) or spexin (14). However, food spitting activity did not exhibit significant changes in our seasonality study or parallel experiments with long-term/short-term acclimation to different temperatures and the involvement of this food rejection behavior in the seasonal cycle of feeding is rather unlikely. In our study, using the fish acclimated to summer temperature (28°C) as a reference, long-term and short-term acclimation to winter temperature (15°C) were both effective in mimicking the decrease in foraging activity and food intake observed during the seasonal change from summer to winter. The results of short-term acclimation (from 28 to 15°C and from 15 to 28°C) also reveal that the changes in feeding responses were highly reversible and rapid modifications in feeding behavior/food intake could be noted within 24 h exposure to temperature change. Our findings are highly comparable with the previous study in salmon parr showing that a short-term cold stress (>4 h) was sufficient to induce a rapid drop in food intake (34) and provide evidence that temperature change in the environment can trigger the seasonal cycle of feeding in goldfish, presumably via a rapid modulation in feeding behavior/foraging activity.

In homeotherms, including birds and mammals, modification of food intake by thermal stress (1, 35) is typically associated with corresponding changes in orexigenic/anorexigenic signals in the brain as well as in peripheral tissues (e.g., GI tract and adipose tissue) (2, 3, 36). In mammals (e.g., rat), the central effects of thermal regulation are commonly accepted to be mediated by the temperature-sensitive neurons within the hypothalamus (37), presumably via activation of thermo-TRP ion channels (38). In bony fish, the functional roles of orexigenic factors including NPY (33), orexin (39), AgRP (40), apelin (41), and ghrelin (42) and anorexigenic factors including CCK (43), CART (44), αMSH (45), MCH (46), and leptin (47) in appetite control are well-documented, but not much information is available for their regulation by temperature change. At present, only four studies have been reported on this topic in fish models. These include the previous studies showing up-regulation of CART in the hypothalamus of Atlantic cod (*Gadus morhua*) at low temperature (6) and reduction in hypothalamic levels of ghrelin receptor and NPY in salmon (*Salmo salar*) with parallel drops in plasma ghrelin at high temperature (11). Recently, two other reports have been published demonstrating that ghrelin and CCK expression in the brain could be elevated by high temperature in perch (*Siniperca chuatsi*) (12) and seahorse (*Hippocampus erectus*) (48), respectively. Unfortunately, the results from these studies are still limited and a common consensus has not been reached for temperature control of feeding based on the feeding regulators examined. In fish models, seasonal variations in central expression of orexigenic/ anorexigenic signals has been reported, e.g., for ghrelin (49), leptin (50), CCK (51), and NPY (52). Therefore, it would be tempting to speculate that their regulation by temperature can mediate the circannual cycle of food intake. However, the idea was not supported by the recent study in Arctic charr (*Salvelinus alpinus*), in which the seasonal patterns of NPY, AgRP, POMC, CART, and leptin expression in brain areas involved in appetite control did not match with its circannual rhythm of feeding (13). To date, the functional link between seasonal cycle of feeding and thermal regulation of orexigenic/anorexigenic signals in the fish brain remains unclear and further studies are highly warranted.

To shed light on the role of orexigenic/anorexigenic signals in seasonal change of feeding in cyprinid species, long-term acclimation of goldfish during the summer at 28°C and during the winter at 15°C were also conducted. In fish models, e.g., salmon (*Salmo salar*) (53), common carp (*Cyprinus carpio*) (54), and more recently in goldfish (*Carassius auratus*) (47), two forms of leptin, namely leptin I and II, have been identified, which are believed to be the result of fish-specific/3R whole genome duplication (55). Unlike mammals with leptin expressed mainly in adipose tissue, leptin is expressed at high levels in the liver of fish species (54–56) and exerts its effect as a satiety factor by regulating central expression of NPY, POMC and/or CCK, e.g., in goldfish (*Carassius auratus*) (57) and trout (*Oncorhynchus mykiss*) (58). When compared with its “summer counterpart” at 28°C, goldfish at 15°C during the winter was found to have notable elevations in leptin I and II mRNA levels in the liver with parallel rises of LepR gene expression in the telencephalon, hypothalamus and optic tectum, which are the major brain areas in goldfish involved in appetite control (7). Although the functional roles of NPY, AgRP, orexin, and apelin as orexigenic factors in fish models are well-documented (59) and their stimulatory effects on feeding have also been confirmed in goldfish (33, 41, 60), except for the drop in orexin mRNA occurring in the hypothalamus at 15°C, noticeable changes in gene expression for these feeding stimulators were not observed in the brain areas examined. In the same study, 15°C acclimation during the winter was found to up-regulate central expression of anorexigenic factors, including the transcript expression of CCK, CART, and POMC in the telencephalon and CCK, MCH, and POMC in the hypothalamus. In contrast, significant changes of leptin I, leptin II, CCK, CART, MCH, and POMC signals were not apparent in the optic tectum. A similar pattern of transcript expression observed in our seasonality study was also noted in our time-course experiment with a gradual drop of water temperature to 15°C within 6 h in goldfish acclimated at 28°C. In this case, similar to the rapid responses of foraging/food intake with short-term thermal acclimation, notable changes of transcript expression for leptin I and II in the liver as well as LepR and other feeding regulators expressed in different brain areas were also observed within 6–12 h exposure to temperature change and maintained up to 24 h during the course of the experiment. These results, as a whole, suggest that the reduction in foraging activity and food intake in goldfish caused by the seasonal change in water temperature may be mediated by the rises of leptin I and II signals in the liver with parallel enhancement in leptin sensitivity via LepR up-regulation in brain areas involved in feeding control. Meanwhile, central regulation of orexigenic/anorexigenic signals can also occur, with a down-regulation of orexin in the hypothalamus along with parallel rises of CCK, CART, MCH, and POMC expression in the telencephalon/hypothalamus. In our study, the orexigenic/anorexigenic factors expressed in the optic tectum did not exhibit seasonal change/noticeable responses to temperature drop. Presumably, this brain area is not a major site within the CNS for temperature sensing or thermal responses in goldfish.

In summary, we have confirmed that seasonal change of feeding with a parallel reduction in foraging activity and food intake do exist in goldfish as a result of temperature drop during the transition from summer to winter period. These feeding responses can occur rapidly and are highly reversible with respect to temperature change, and may involve the leptin output from the liver with differential modifications of orexigenic/anorexigenic signals and leptin responsiveness in brain areas for appetite control. To our knowledge, our study represents the first report on (i) thermal regulation of leptin expression in the liver and (ii) involvement of leptin/LepR system in seasonal change of feeding induced by temperature drop in a fish model. Although our studies have provided new insights on the mechanisms for seasonal change of feeding in fish species, the functional components for thermal detection, e.g., through the thermal sensing neurons within the hypothalamus (37) or vagus nerve network (61), have yet to be determined. Of note, POMC expression induced by CCK (43) and CART expression induced by leptin (62) and MCH (63) have been reported in goldfish, especially in brain areas responsible for feeding control, and the possibility for functional interactions among different feeding regulators in the seasonal cycle of feeding cannot be excluded. Besides temperature change, photoperiod is another environmental cue known to affect feeding in fish species (4). Given that melatonin has been shown to inhibit food intake in goldfish (64) and stimulate leptin expression in the liver (65) with parallel changes of orexigenic/anorexigenic signals in the brain of zebrafish (*Danio rerio*) (66), the functional interplay between photoperiod and temperature via a “crosstalk” of melatonin with other feeding regulators for sure can be an interesting topic to follow up for the seasonal change of feeding in fish models.

## Data Availability

The raw data supporting the conclusions of this manuscript will be made available by the authors, without undue reservation, to any qualified researcher.

## Ethics Statement

The study was carried out in accordance with the recommended guidelines for the care and use of laboratory animals for research and teaching at the University of Hong Kong (Hong Kong). The protocol used in our study (CULATR 4608-18) was approved by the Committee on the Use of Live Animal for Teaching and Research, University of Hong Kong.

## Author Contributions

AW was the PI and grant holder. AW and TC were responsible for project planning and data analysis. TC and MW were involved in the experiments for seasonal studies and thermal acclimation. Manuscript preparation was done by AW and BC.

### Conflict of Interest Statement

The authors declare that the research was conducted in the absence of any commercial or financial relationships that could be construed as a potential conflict of interest.

## Abbreviations

AgRP: Agouti-Related Peptide
α-MSH: Alpha Melanocyte-Stimulating Hormone
CART: Cocaine- and Amphetamine-Regulated Transcript
CCK: Cholecystokinin
MCH: Melanin-Concentrating Hormone
NPY: Neuropeptide Y
POMC: Proopiomelanocortin
LepR: Leptin Receptor
CNS: Central Nervous System.
